# Supplementary material for: The genome sequence of the tub gurnard, Chelidonichthys lucerna (Linnaeus, 1758) (Perciformes: Triglidae)
Source: Wellcome Open Res. 2026 Mar 18;11:12. Originally published 2026 Jan 6. [Version 2] doi: 10.12688/wellcomeopenres.25417.2 (PMC12856256; doi:10.12688/wellcomeopenres.25417.2)
Supplement: Peer review history

# Reviewer report: Reviewer response for version 2

**DOI:** 10.21956/wellcomeopenres.28685.r151043

**Published:** 2026-03-20

**Authors:** Bingpeng Xing

**Affiliations:** 1 Third Institute of Oceanography Ministry of Natural Resources, Xiamen, Fujian, China

The revised manuscript has satisfactorily addressed the major issues raised in the previous round, particularly the incorrect species information in the Background section, which has now been corrected. The genome assembly remains of high quality, and the overall manuscript has improved substantially.  A few minor issues still remain, including an inconsistency in the species name spelling ( *Chelidonichthys lucerne*?), insufficiently explicit explanation of k-mer completeness, and the need for clarification of how sequencing coverage was calculated. These are all minor textual issues.

Are sufficient details of methods and materials provided to allow replication by others?

Yes

Is the rationale for creating the dataset(s) clearly described?

Yes

Are the datasets clearly presented in a useable and accessible format?

Yes

Are the protocols appropriate and is the work technically sound?

Yes

Reviewer Expertise:

marine biodiversity；DNA barcoding；genome；marine ecosystems；mangrove

I confirm that I have read this submission and believe that I have an appropriate level of expertise to confirm that it is of an acceptable scientific standard.

# Reviewer report: Reviewer response for version 1

**DOI:** 10.21956/wellcomeopenres.28003.r144346

**Published:** 2026-01-29

**Authors:** Ivan Rodrigo Wolf

**Affiliations:** 1 UNC Charlotte Department of Biological Sciences, Charlotte, North Carolina, USA

The manuscript "The genome sequence of the red gurnard, *Chelidonichthys lucerna* (Linnaeus, 1758) (Perciformes: Triglidae)" describes the genome assembly of *Chelidonichthys lucerna*. This fish is widely distributed in the eastern Atlantic and adjacent seas (Mediterranean and Black Sea), inhabiting depths ranging from 20 to 250 meters.

 As part of Darwin Tree of Life Project, the methods follow a series of standardized protocols for DNA and RNA extraction and preparation for sequencing at the Wellcome Sanger Institute (WSI). The specimen used to obtain biomolecules was identified by a specialist and confirmed by DNA barcoding. In addition, the bioinformatics protocols utilize a set of current and classic softwares for the assembly and curation of *C. lucerna* haplotypes.

 The assembly quality metrics are excellent compared to the benchmarks, with only the completeness of the *k*-mers being below expectations. Unfortunately, the data still lacks robust annotations, but the RNA-Seq data is publicly available.

Are sufficient details of methods and materials provided to allow replication by others?

Yes

Is the rationale for creating the dataset(s) clearly described?

Yes

Are the datasets clearly presented in a useable and accessible format?

Yes

Are the protocols appropriate and is the work technically sound?

Yes

Reviewer Expertise:

Bioinformatics, systems biology, genomics, transcriptomics, molecular phylogenetics, immunogenomics, pattern recognition, search and characterization of genes of biotechnological interest.

I confirm that I have read this submission and believe that I have an appropriate level of expertise to confirm that it is of an acceptable scientific standard.

# Reviewer report: Reviewer response for version 1

**DOI:** 10.21956/wellcomeopenres.28003.r144349

**Published:** 2026-01-13

**Authors:** Bingpeng Xing

**Affiliations:** 1 Third Institute of Oceanography Ministry of Natural Resources, Xiamen, Fujian, China

This manuscript presents a high-quality chromosome-level genome assembly of Chelidonichthys lucerna generated using state-of-the-art PacBio HiFi and Hi-C technologies. The assembly metrics meet or exceed Earth BioGenome Project reference standards.

 However, 1)there is a critical species-name inconsistency in the Background section ( *C. cuculus *vs *C. lucerna*), and several areas would benefit from clearer explanation of haplotype completeness and assembly interpretation.  

 2)The full binomial ( *Chelidonichthys lucerna*) should be used at its first occurrence, and the abbreviated form ( *C. lucerna*) may be used thereafter.

 3)Explicitly state the intended use of haplotype 2 ( for phasing completeness rather than downstream analyses). Clarify that reduced k-mer completeness for individual haplotypes is an expected outcome of haplotype-resolved assembly, not an indicator of reduced assembly quality.

 4)The reported ~127× genome coverage is appropriate but it is not entirely clear whether this refers to raw HiFi read coverage or post-filtering effective coverage.

Are sufficient details of methods and materials provided to allow replication by others?

Yes

Is the rationale for creating the dataset(s) clearly described?

Yes

Are the datasets clearly presented in a useable and accessible format?

Yes

Are the protocols appropriate and is the work technically sound?

Yes

Reviewer Expertise:

marine biodiversity；DNA barcoding；genome；marine ecosystems；mangrove

I confirm that I have read this submission and believe that I have an appropriate level of expertise to confirm that it is of an acceptable scientific standard.
